# Social Drinking on Social Media: Content Analysis of the Social Aspects of Alcohol-Related Posts on Facebook and Instagram

**DOI:** 10.2196/jmir.9355

**Published:** 2018-06-22

**Authors:** Hanneke Hendriks, Bas Van den Putte, Winifred A Gebhardt, Megan A Moreno

**Affiliations:** ^1^ Amsterdam School of Communication Research University of Amsterdam Amsterdam Netherlands; ^2^ Trimbos Institute Netherlands Institute for Mental Health and Addiction Utrecht Netherlands; ^3^ Health, Medical and Neuropsychology Unit Institute of Psychology Leiden University Leiden Netherlands; ^4^ Department of Pediatrics University of Wisconsin Madison Madison, WI United States

**Keywords:** social media, alcohol drinking, social interaction

## Abstract

**Background:**

Alcohol is often consumed in social contexts. An emerging social context in which alcohol is becoming increasingly apparent is social media. More and more young people display alcohol-related posts on social networking sites such as Facebook and Instagram.

**Objective:**

Considering the importance of the social aspects of alcohol consumption and social media use, this study investigated the social content of alcohol posts (ie, the evaluative social context and presence of people) and social processes (ie, the posting of and reactions to posts) involved with alcohol posts on social networking sites.

**Methods:**

Participants (N=192; mean age 20.64, SD 4.68 years, 132 women and 54 men) gave researchers access to their Facebook and/or Instagram profiles, and an extensive content analysis of these profiles was conducted. Coders were trained and then coded all screenshotted timelines in terms of evaluative social context, presence of people, and reactions to post.

**Results:**

Alcohol posts of youth frequently depict alcohol in a positive social context (425/438, 97.0%) and display people holding drinks (277/412, 67.2%). In addition, alcohol posts were more often placed on participants’ timelines by others (tagging; 238/439, 54.2%) than posted by participants themselves (201/439, 45.8%). Furthermore, it was revealed that such social posts received more likes (mean 35.50, SD 26.39) and comments than nonsocial posts (no people visible; mean 10.34, SD 13.19, *P*<.001).

**Conclusions:**

In terms of content and processes, alcohol posts on social media are social in nature and a part of young people’s everyday social lives. Interventions aiming to decrease alcohol posts should therefore focus on the broad social context of individuals in which posting about alcohol takes place. Potential intervention strategies could involve making young people aware that when they post about social gatherings in which alcohol is visible and tag others, it may have unintended negative consequences and should be avoided.

## Introduction

### Alcohol and Youth

Alcohol consumption and binge drinking among adolescents and young adults have been consistently linked with negative consequences such as accidents, harassment, vandalism, sexual abuse, impaired brain development, and disease [1-3]. Alcohol abuse is therefore regarded as a major cause of preventable death and morbidity [4]. Many young people, however, consume alcohol frequently and often excessively. Recent studies in Europe and the United States showed that 35% of youth who consume alcohol had at least 1 binge drinking episode (ie, drinking 5 or more drinks on 1 occasion [5]) in the past month [5-7]. This high prevalence of alcohol use and the multitude of negative consequences increase the need to gain more insight into the context in which this behavior takes place. Research has shown that alcohol is often consumed in social contexts [8]. An emerging social context in which alcohol is becoming increasingly apparent is that of social media. Young people increasingly display alcohol-related posts on social networking sites such as Facebook and Instagram [9]. This study investigated those alcohol posts on social media, and how social those posts actually were, by conducting a content analysis of alcohol posts on Instagram and Facebook among 192 adolescents and young adults.

### Social Context of Alcohol

Young people often consume alcohol in social contexts such as dinners and parties, and alcohol consumption is often referred to as a social activity [8,10,11]. Research has shown that alcohol consumption plays a large part in young people’s social lives and social identity exploration [12]. Furthermore, many studies have shown that social norms are strongly related to adolescent alcohol use, especially when these norms originate from peers [2]. The importance of the social environment for alcohol consumption is also apparent from the vast amount of research demonstrating the effects of peer influence and social networks on alcohol consumption [13-16]. Rosenquist et al [17], for example, showed that when close friends within a person’s social network drink alcohol, this greatly increases the chance that the person drinks alcohol as well. Taken together, alcohol use should be seen as part of the social context in which it is so strongly embedded.

### Alcohol and Social Media

Recent technological changes have drastically changed the way in which young people shape their social lives, and new social contexts have emerged as a consequence. Adolescents and young adults increasingly spend their time communicating with others in online settings [18,19]. Social networking sites such as Facebook and Instagram play an important role in young people’s daily lives, accounting for a large portion of their time [20-22]. Research has shown that these social networking sites are frequently used by young people to communicate about alcohol, and multiple studies have demonstrated that many adolescents and young adults (percentages vary between 36% and 96%) display alcohol-related posts on Facebook (eg, photos in which young people hold alcoholic beverages [9,23-24]).

Alarmingly, studies have found positive associations between alcohol posts on social media and self-reported alcohol consumption. For example, Moreno et al [25] found that posting about alcohol use on Facebook was associated with increased offline drinking behavior. Similarly, Boyle et al [26] observed that exposure to alcohol posts on Facebook, Instagram, and Snapchat predicted more alcohol consumption 6 months later (see also Geusens and Beullens [27]). Thus, there is evidence suggesting a relation between both posting and being exposed to alcohol posts on social media and actual drinking behavior.

A few studies have examined the content of alcohol posts in more detail, usually by employing a clinical framework to evaluate individuals’ alcohol posts. These studies have found that alcohol posts suggesting problematic alcohol use are more exceptional than posts showing regular use (ie, posts about alcohol but not about problem drinking or intoxication) but are more predictive of alcohol abuse [9,23-24,28-29]. However, these studies focus on the individual rather than the individual in a social context, and in-depth knowledge on the social aspects of these posts is lacking. Considering the importance of social aspects for both alcohol consumption and social media use, this study aims to provide insight into the social content of alcohol posts (ie, the evaluative social context and the presence of people) and the social processes (ie, the posting of and reactions to posts) involved with alcohol posts on social networking sites.

### Social Content of Alcohol Posts

Alcohol consumption and social media use are inherently social [10]. Although some studies have emphasized the social nature of alcohol posts [12,30], few studies to date have focused on the exact social content displayed in these posts. Having more insight on the extent to which and how the content of alcohol posts are social can greatly increase our understanding of alcohol-related social media use and provide valuable information for intervention strategies (eg, whether an individualistic or social intervention strategy would work better). In this study, we examined 2 social aspects of alcohol-related content: the social evaluative context toward alcohol (ie, whether alcohol is portrayed in a negative, neutral, or positive context) and the presence of people in a post (eg, whether alcohol posts show a close-up of a cocktail, display 1 person drinking wine, or depict groups of people at a party drinking beers). With regard to the social evaluative context, research has shown that the valence of social interactions about alcohol can determine conversation effects [31,32]. For example, Hendriks et al [33] showed that when interpersonal interactions are positive toward alcohol, this can lead to increased alcohol consumption, and when conversations are mainly negative toward alcohol, this can reduce drinking behaviors. It is therefore important to illuminate the social evaluative context of alcohol-related content on social media. In this study, we investigated whether alcohol posts on social media show a negative social context (eg, someone looking disapprovingly at a drunk person) or positive social context (eg, people toasting and laughing), in line with research by Beullens and Schepers [23].

With regard to the presence of people in a post, it has been found that people are easily persuaded to engage in a specific behavior merely by observing other people portraying that behavior. Examples of such studies range from recent experiments showing that healthy social norms in health campaigns (eg, many people in a health ad displaying healthy conduct) can encourage the uptake of healthy behaviors [34-36] to classic studies by Asch [37] demonstrating the powerful impact of other people on participants’ willingness to engage in similar behaviors. In addition, there are indications that the social conformity effects found by Asch [37] are a function of group size [38]. These findings are in line with the idea of a basic human need to belong—people have a strong need to fit in with a group [39]. Given this evidence, we investigated the extent to which people are displayed in alcohol posts. We pose the following research questions about social content (RQ1):

RQ1a: Do alcohol posts reflect a negative or positive social evaluative context?

RQ1b: To what extent are people present in alcohol posts?

### Social Processes Involved With Alcohol Posts

In addition to the social content of alcohol posts, it is also important to understand the social processes involved with the posting of alcohol-related content on social media because this can further enhance our understanding of the social nature of alcohol posts. Two factors related to social processes are explored in this study: how alcohol posts get posted and how people respond to these posts. First, it is unclear whether people actively post about alcohol themselves or whether this is part of a social process in which they are tagged (mentioned) in posts by others. Whether people actively post about alcohol or are passively tagged in alcohol posts is important because research has suggested that active (ie, talking) versus passive (ie, listening) interpersonal communication can lead to different effects of this communication. For example, Janis and King [40] asked people to either deliver a speech advocating for a certain issue or listen to that same speech and showed that people who talked were more persuaded by the speech than people who listened. Who posts alcohol posts is relevant to understand for practical reasons because knowing this can provide important information for future interventions aiming to decrease the posting of such content or its negative impact on the individual. If people are mostly tagged in posts by others this requires a different intervention strategy (by encouraging tagged people to ask to be removed or not included in the alcohol post) than if people post about alcohol themselves (by directly discouraging people to post about alcohol).

Second, it is not yet clear whether and how negatively or positively others respond to alcohol posts on social media. Do alcohol posts receive likes and are comments supportive of the posts? Research has revealed that approval of a behavior (ie, a supportive injunctive norm) encourages the behavior [34,36]. Likes (and supportive comments) on social media can illustrate such a social norm and are therefore important factors potentially determining a post’s influence. This was suggested by Alhabash et al [41], who investigated the effects of alcohol posts and found that posts with many likes had especially strong persuasive effects. It is therefore important to provide insight into the reactions that alcohol posts trigger.

An additional relevant question addressed in this study is whether reactions to alcohol posts depend on the social content of the post. For example, do alcohol posts with people in them get more likes than posts with no people in them, and do posts with a positive social evaluative context receive more supportive comments than less positive social posts? This seems likely given the abovementioned studies on the importance of social norms and the need to belong [34,39]; alcohol posts showing people may portray alcohol-supportive norms, especially when people are holding alcoholic beverages, that could cause viewers to behave in line with their need for belongingness and approve of such norms by giving supportive reactions. By liking social alcohol posts, people can express their feelings of friendship and need to belong to the people depicted in the pictures. We pose the following research questions about social processes related to alcohol posts (RQ2):

RQ2a: Who posts alcohol posts (do participants themselves post or are participants tagged)?

RQ2b: What are the responses of others to alcohol posts?

RQ2c: Do responses to alcohol posts depend on whether the content of the post is social?

In sum, this study aimed to provide insight into the social content of alcohol posts and the social processes involved with alcohol posts on social networking sites. This aim was addressed through a content analysis of alcohol posts on Facebook and Instagram.

## Methods

### Participants and Design

This study was part of a larger data collection during which 561 participants filled out a questionnaire regarding social media use (questionnaire data are not the focus of this study). For the purposes of this paper, participants were asked whether we could access (friend) their Instagram and Facebook profiles, and 214 of these participants gave their consent to do so. Of these participants, 22 were excluded from later analyses because they did not fall into the intended age category (12 to 30 years), resulting in a total of 192 participants to be analyzed (mean 20.64, SD 4.68 years, 132 women and 54 men). Due to technical problems, 6 participants could not be successfully linked to their questionnaire data. Their profiles could be coded, however, and are included in the analyses. Of the participants (N=192), 106 had only a Facebook profile, 15 had only an Instagram profile, and 70 had both.

### Procedure

The participants who agreed to give access to their profiles were asked to accept a friend request from a research profile on Facebook and/or Instagram, allowing all participant posts to be accessed by the researchers. Participants were informed that all data would be stored anonymously; all personal information (ie, names and faces) would be removed from the posts so that these could not be traced back to the participants. Screenshots were made of all timelines on profiles for the period of the previous year (April 2015 to April 2016), after which friendships on Facebook and Instagram were cancelled and the research profile was deleted. For participants younger than 18 years, consent was required from the parents as well as the adolescents. This study was approved by the university’s ethics committee.

### Content Analysis

#### Coder Training

Two coders were trained in 3 sessions led by the first author, during which several example profiles were coded and inconsistencies were discussed. After the training was finished, 10% of the profiles were coded in order to assess coder reliability, after which all profiles were coded. Coder agreement was acceptable (kappa=.683-.912). Please note that we could not calculate agreement for variables with a low *n* (eg, posts with a negative social evaluative context or Instagram posts).

#### Coding Procedure

A codebook described the coding process. Coders were asked to scroll down the timeline for the past year and to look at each post. Once an alcohol post was identified, several variables were recorded in Excel (Microsoft Corp). The profiles were coded by 1 of 2 coders. When coders were unsure how to code a post, they discussed it with the first author after which a choice for coding was made.

#### Coding Variables

##### Occurrence and Frequency

An alcohol post was defined as “a post about alcohol or in which alcohol is visible.” Coders were asked to take the whole post (ie, the photo/video including headings/texts) into account. Coders coded whether there were any alcohol posts visible (occurrence: no/yes) on the profile, and if so, how many they identified on the profile (frequency). If no alcohol posts were present, no further information for that profile was needed. If an alcohol post was identified, however, the coder was asked to code the additional variables (see below). Most Facebook alcohol posts (410/442, 92.8%) consisted of a photo accompanied by a caption; 7.2% (32/442) consisted solely out of text.

##### Social Evaluative Context

Coders were asked to describe whether the context was negative, neutral, or positive toward alcohol. Social evaluative context was based on Beullens and Schepers [23]; in line with their coding book, we coded whether an alcohol post showed a negative context (eg, someone looking disapprovingly at a drunk person), a neutral context (eg, no explicit judgment or emotion is shown), or a positive context (eg, people laughing and toasting with alcoholic drinks). The context could also be inferred from a caption (eg, when a photo showed a close-up of a dinner table including a glass of wine with the caption “Having a lovely time!”). Text-only posts were coded in a similar manner: negative if alcohol was described with negative words (“drank too much; headache!”), positive if alcohol was described with positive words (“I’m looking forward to boozing tonight!”).

##### People Present in Post

**Figure 1:**
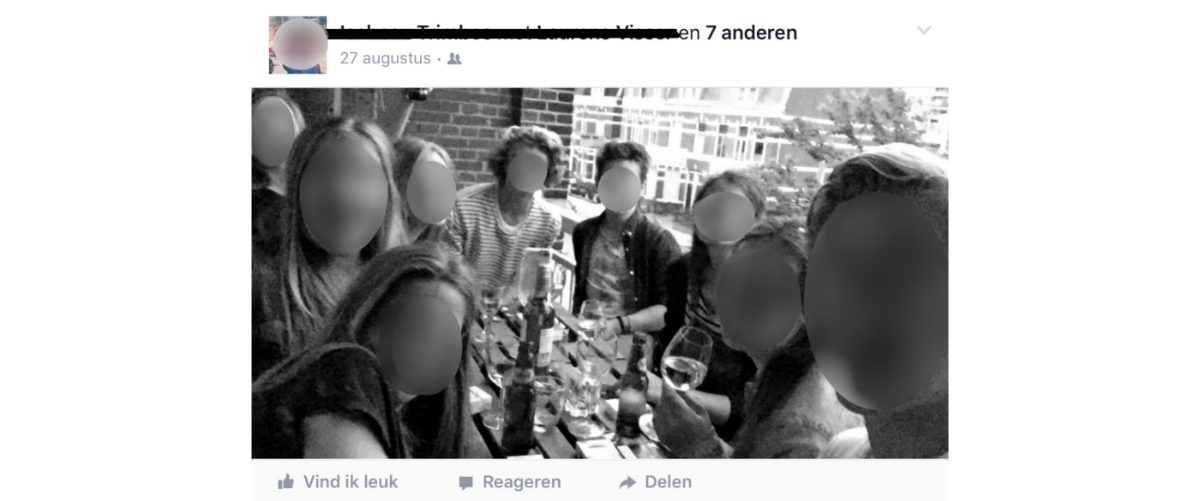
Example of a post that would be coded as having a positive evaluative context, showing the participant with others, and showing the participant holding a drink.

**Figure 2:**
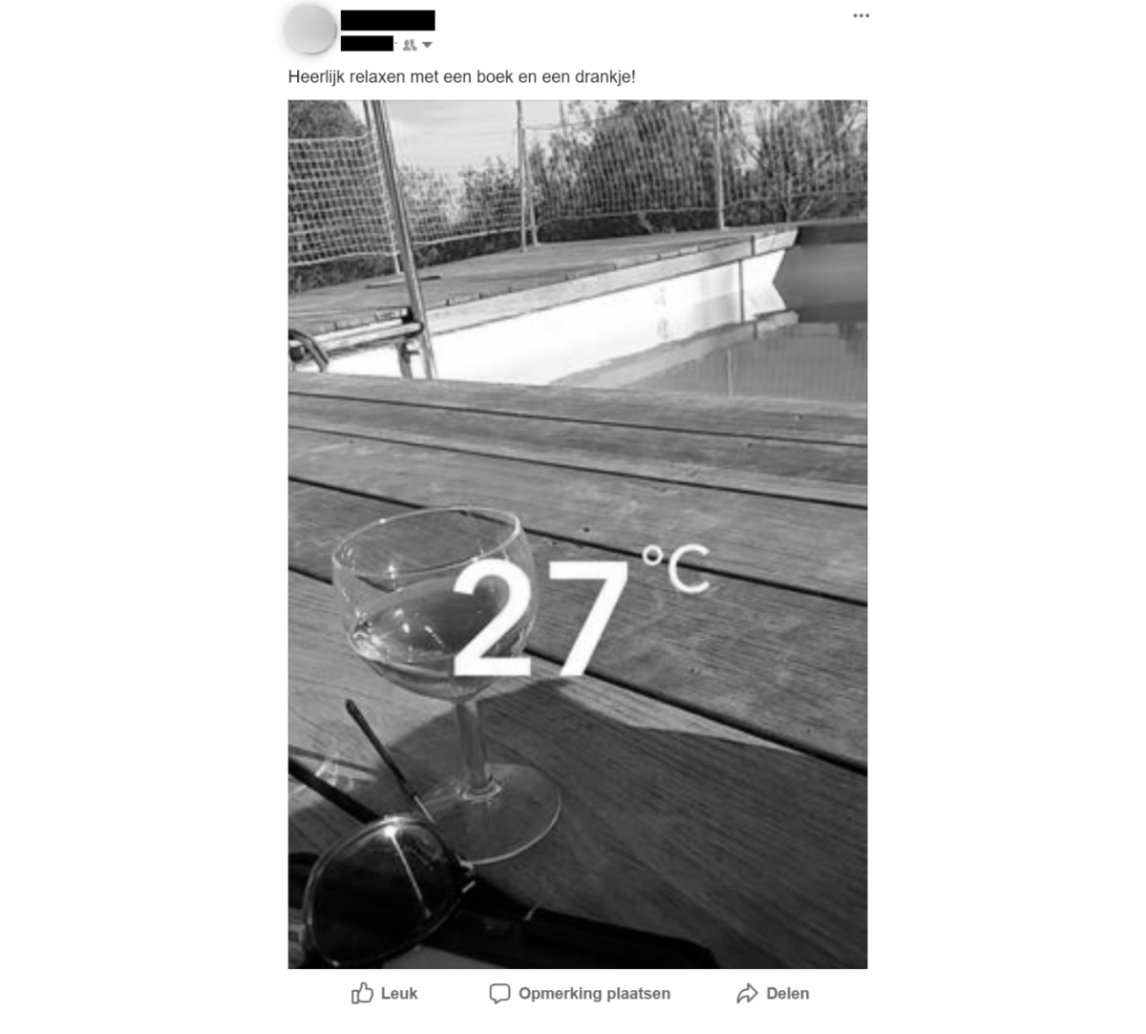
Example of a post that would be coded as having a positive evaluative context and no people visible. The caption translates as "Lovely relaxing time with a book and drink!".

Given research [34-39] showing that the presence of people can affect persuasion, we coded whether people were present in the post (no people visible/tagged [text-only posts], participant only, others only, or participant with others). We also coded whether someone was holding an alcoholic beverage, and if so, who (no one, participant, others). The reason we measured the latter variable is because holding a beverage can be seen as a clear indication of descriptive norms (person is drinking) as well as injunctive norms (person must like drinking [42]). Figures 1 and 2 show examples of how a post would be coded in terms of evaluative context and presence of people.

##### Placer of Post

We coded whether the post was placed by the participant or by others (with participant tagged) based on previous studies suggesting different effects of active versus passive interpersonal communication [40].

##### Reactions to Posts

We coded how many likes and comments the post received and whether the comments were mainly negative (“Pathetic!” or “You’re not looking too well”), neutral (“Where is this?” or “What are you drinking?”), or positive (“Nice shot!” or “Looking good!”) toward the participants and/or post. Valence of comments was adjusted from Beullens and Schepers [23].

### Data Analysis

To investigate the social content (RQ1a/RQ1b) and processes (RQ2a/RQ2b) involved with alcohol posts we first described the frequencies and descriptives of the coded variables. In order to address whether the likes and comments on alcohol posts depend on the content of the posts (RQ2c), we conducted analyses of variance with the social content of the post as independent variable (presence of people, holding of beverages, and placer of post) and likes, comments, and valence of comments as dependent variables. 

Because *n* was too low, we did not investigate the influence of a negative (2 Facebook posts) versus positive (11 Facebook posts) context on reactions, and we decided not to analyze Instagram posts (posted by only 24 participants). We consider differences with *P*<.01 as significant in order to compensate for multiple comparisons [43].

## Results

### Occurrence and Frequency of Alcohol Posts

Results showed that 51.1% (90/176) of participants with a Facebook profile had at least 1 alcohol post on their profile during the past recorded year. On Instagram, 28% (24/85) of participants had a profile with at least 1 alcohol post. The participants who had an alcohol post on their Facebook profile posted on average 5 alcohol posts during the past recorded year (mean 5.02, SD 6.32). On Instagram, among the participants who posted an alcohol post, the average number of alcohol posts was 4 (mean 3.91, SD 4.21).

The following section focuses solely on the alcohol-related posts by participants on Facebook (n=90) or on Instagram (n=24). Participants with Facebook or Instagram profiles who posted about alcohol (20 men, 73 women, mean age 21.92, SD 3.94 years) differed slightly from those who did not post about alcohol (34 men, 59 women, mean age 19.35, SD 5.01 years) in terms of age (F_1,184_=15.12, *P*<.001) but not significantly in terms of gender (χ^2^_1_=5.1, *P*=.02). In total, we analyzed 442 alcohol posts on Facebook and 94 alcohol posts on Instagram.

Please note that the total number of posts described sometimes varies—the total number of posts mentioned at social evaluative context is 438, whereas the total number of posts mentioned at placer of post is 439 because coders sometimes had to code a variable as missing (eg, when the context of the post could not be inferred or when the valence of comments could not be coded because there were none). The percentages shown are based on the total number of posts that were coded for that variable.

### RQ1: Social Content

#### Social Evaluative Context

The majority of the alcohol posts on Facebook depicted alcohol in a positive context (425/438, 97.0%), a few showed a neutral context (11/438, 2.5%), and almost none depicted a negative context (2/438, 0.5%). On Instagram, these percentages were similar (see Table 1).

#### Presence of People

Most Facebook alcohol posts (277/412, 67.2%) displayed a social context with other people visible in the posts, but 16.3% (67/412) of alcohol posts did not show any people. Similar patterns were seen on Instagram (Table 1). Beverages were held by participants as opposed to appearing on a table. Thus, on both platforms, most alcohol posts displayed a social context with the participant and other people shown.

### RQ2: Social Processes

#### Placer of Post

Alcohol posts were often placed by participants themselves on Facebook (201/439, 45.8%), but, more frequently, the posts were placed by others (238/439, 54.2%) with the participant tagged (Table 2). Tagging was not yet possible on Instagram in 2015.

#### Reactions to Alcohol Posts

On average, an alcohol post on Facebook received 29 likes (mean 29.67, SD 26.15) and 3 comments (mean 3.41, SD 5.36). Almost all alcohol posts on Facebook got at least 1 like (421/442, 95.9%), and most got at least 1 comment (309/442, 71.9%). This was similar on Instagram (see Table 2).

Although not all alcohol posts received comments; of those that did, the responses were mostly positive (243/297, 81.8%) toward the post and/or participant (neutral: 49/297, 16.5%; negative: 5/297, 1.7%). The pattern was similar on Instagram (see Table 2).

### RQ2c: Do the Responses to Alcohol Posts Depend on the Content of the Post?

#### Presence of People

First, there was a significant effect of presence of people (no one vs only participant vs only others vs participant together with others) on the number of likes (*F*_3,406_=18.96, *P*<.001). Posts without any people present in them yielded significantly fewer likes (mean 10.34, SD 13.19) than posts in which the participant was shown alone (mean 30.63, SD 21.21, *P*=.002), posts in which only others were displayed (mean 25.71, SD 28.98, *P*=.001), or posts in which others were shown together with the participant (mean 35.50, SD 26.39, *P*<.001). Posts in which others were shown together with the participant yielded more likes than posts with only others (*P=*.01). Posts without any people present resulted in significantly fewer comments (mean 2.22, SD 3.20) than posts displaying others without the participant (mean 4.47, SD 7.13, *P*=.01). Posts in which the participant was alone (mean 3.68, SD 5.25) did not differ from the other posts in terms of comments (*P*>.22). The valence of comments was not influenced by presence of people (*F*_3,267_=0.47, *P=*.70). Thus, social posts yielded more responses and more positive reactions than posts without people in them.

#### Holding of Beverages

A significant effect of holding of beverages (no one versus participant versus others) on likes was revealed (*F*_2,322_=14.36, *P*<.001). That is, posts without someone explicitly holding an alcoholic beverage resulted in significantly fewer likes (mean 19.20, SD 23.86) than posts in which the participant (mean 30.94, SD 22.97, *P*=.001) and/or others (mean 37.34, SD 29.94, *P*<.001) were holding drinks in their hands. There were no significant effects on the number or valence of comments, all *F*<0.92, all *P*>.40.

#### Placer of Post

A significant effect of placer of post (participant vs others) on likes was found (*F*_1,434_=8.50, *P*=.004). That is, posts placed by others yielded significantly more likes (mean 33.02, SD 25.05]) than posts placed by the participants themselves (mean 25.77, SD 26.80). There were no significant effects on the number or valence of comments (all *F*<1.55, all *P*>.21). Thus, in general, more social posts (eg, in which others are present, which are posted by others, and in which people hold drinks in their hands) resulted in more reactions, in particular more likes. For an overview of the effects of social content on likes, see Table 3.

**Table 1:** Social content of alcohol-related posts on Facebook and Instagram.

Variable		Facebook (n=442), n (%)	Instagram (n=94), n (%)
**Social evaluative context**			
	Negative	2 (0.5)	0 (0)
	Neutral	11(2.5)	0 (0)
	Positive	425 (96.1)	94 (100)
**People present**			
	No one	67 (16.3)	28 (29.8)
	Only participant	19 (4.6)	10 (10.6)
	Participant and others	277 (67.2)	49 (52.1)
	Only others	49 (11.9)	7 (7.4)
**Beverages in hand**			
	No one	116 (35.4)	25 (43.1)
	Participant	98 (29.9)	19 (32.8)
	Others	114 (34.8)	14 (21.4)

**Table 2:** Social processes involved with the posting of alcohol-related posts on Facebook and Instagram.

Variable		Facebook (n=442)	Instagram (n=94)
**Placement of post, n (%)**			
	Participant	201 (45.8)	94 (100)
	Others	238 (54.2)	—
**Valence of post reactions, n (%)**			
	Negative comments	5 (1.7)	1 (2.3)
	Neutral comments	49 (16.5)	6 (13.6)
	Positive comments	243 (81.8)	37 (84.1)
**Number of post reactions, mean (SD)**			
	Number of likes	29.67 (26.15)	43.12 (52.11)
	Number of comments	3.41 (5.36)	3.82 (3.01)

**Table 3:** Significant differences in likes of alcohol-related posts on Facebook based on social content.

Variable		Number of likes on Facebook		
		Mean (SD)		Median
**People present**				
	No one	10.34 (13.19)		5.00
	Only participant	30.63 (21.21)		27.00
	Participant with others	35.50 (26.39)		32.00
	Only others	25.71 (28.98)		18.00
**Beverages in hand**				
	No one	19.20 (23.86)		11.00
	Participant	30.94 (22.97)		25.00
	Others	37.34 (29.94)		34.00
**Placement of post**				
	Participant	25.77 (26.80)		18.00
	Others	33.02 (25.05)		28.50

## Discussion

### Principal Findings

The goal of this research was to investigate the social content (ie, evaluative social context and presence of people) and social processes (ie, posting of and reactions to posts) involved with alcohol posts on social networking sites. Two main findings were revealed: in terms of content and processes, alcohol posts on social media are social in nature and these social aspects are related to reactions—the more social elements to the post, the more (positive) reactions the post receives.

The first main finding that alcohol posts are social in terms of content was illustrated in 2 ways. First, alcohol posts showed alcohol in a positive social context, with people approving of the drinking behaviors pictured in the post (eg, by laughing or toasting). This is in line with Beullens and Schepers [23], who found that the context of alcohol consumption on social media is often positive. This approval by others of alcohol consumption in alcohol posts is alarming, as ample studies show that a positive injunctive social norm (the perception that others approve of this conduct) leads to increased drinking behaviors [35,36,42]. Alcohol posts may thus, by enhancing positive social norms, lead to more excessive alcohol consumption. This idea was also suggested in a recent study about cigars and cigarillo images on Instagram in which it was observed that these images are relatively common and could add to the normalization of tobacco in everyday life [44]. More research is needed to investigate this potential mediating effect of social media posts on norms and subsequent drinking behavior.

In addition, alcohol posts are social in the sense that most reflect a social setting (eg, dinners or parties) that display (groups of) people. These groups of people can potentially further increase the effect of the portrayed positive social norms by showing that many approve of drinking alcohol and display this behavior themselves (eg, by holding alcoholic beverages) thereby enhancing descriptive norms (ie, the perception that many others engage in this behavior [42]). This suggests a strong impact of alcohol posts, especially when many people appear in the image. Furthermore, it is possible that positive injunctive norms are especially triggered by a positive social context in alcohol posts and that positive descriptive norms are more strongly affected by whether people are holding these beverages in the picture. Given the importance of social norms for alcohol consumption, this role of social norms within the effects of alcohol posts should be investigated in more detail.

Not only are alcohol posts social in terms of content, they are also social in terms of the processes involved with posting them. That is, alcohol posts receive many likes and comments, and these comments are mostly positive about the post. This is consistent with our findings regarding the social evaluative context: people from the social network appear strong in their approval of alcohol posts. This is important to keep in mind when addressing the issue of alcohol posts; they seem to be posted in a very proalcohol context. Furthermore, this study is the first to reveal that tagging plays an important role in the posting of alcohol-related posts. Many participants had alcohol posts on their profile that they did not post; they had simply been tagged in these posts. This tagging has very relevant implications for interventions aiming to decrease alcohol posts. An individualistic approach (eg, intervention planners asking people to not post an alcohol post on their profile) may not be sufficient. Thus, reducing these tagged posts may require a different strategy by taking into account the social environment (eg, intervention planners asking people to deny tagging, or by asking people not to tag others in such posts).

The second main finding is that social aspects of alcohol posts are related to the responses to these posts. Posts that displayed people, posts in which others were holding drinks, and posts posted by others yielded more likes and often more comments than posts without people in them, posts in which no one was holding a drink, or posts posted by the participants themselves. This further strengthens the idea that alcohol posts are part of a social process, in which the social aspects common to alcohol posts trigger social interactions that show further appreciation of the post and help spread the message even further. Because often-liked posts are featured higher in Facebook’s newsfeed, this can further increase the chance the post will get liked, commented on, or shared.

An explanation for why social posts triggered more, and more positive, interpersonal communication could be that human beings have a strong need to connect with and belong to groups [39]. Many studies have shown the preference that humans have for social stimuli (eg, faces) over nonsocial stimuli (eg, geometrical shapes [45,46]). That social posts get liked more seems therefore to be in line with this fundamental human need for belongingness. The question, however, is how generic or specific this effect is. Do all posts with people in them or posted by others receive more likes and comments? Or is this only, or especially, the case with alcohol consumption, a very social behavior? As this study does not compare alcohol posts with posts regarding other unhealthy behaviors, this remains a question. Research exists, however, showing that young people also post about other health behaviors such as physical activity, snacking, smoking, marijuana use, waterpipe use, and sexual behaviors [44,47-51]. Although they do not always depict groups of people, some of these posts elicit a lot of responses when posted in like-minded fora, such as posts related to “fitspiration” (ie, a recent social media trend designed to motivate people to eat healthily and exercise [52]). Thus, it appears that social aspects of a post are not the only factor influencing post responses. Some recent research suggests that certain posts (such as certain types of active selfies [53]) trigger more responses by increasing narrative involvement. It is possible that the social setting often visible in alcohol posts may also increase involvement in the narrative (eg, being part of a dinner or party) and may therefore lead to more likes and comments. The processes through which social alcohol posts lead to more reactions and whether these processes are distinct for alcohol posts is an important avenue for future studies.

Taking these findings together suggests that alcohol posts on social media are a part of young people’s everyday social lives, in which drinking at dinners or parties and posting and tagging about these social events go hand in hand. This normalcy of alcohol posts not only strengthens the idea in young people’s minds that alcohol is normal and a part of daily life [54,55] but also increases the idea that a lot of people are positive about alcohol and consume alcohol regularly. This can increase pluralistic ignorance (ie, people incorrectly assuming that many people engage in a specific behavior such as alcohol abuse [56]) and lead to more drinking behaviors (in line with Moreno et al [25] and Boyle et al [26]). Although a lot of research exists showing that alcohol use in traditional media (movies or commercials [57-59]) can increase alcohol use of viewers, recent evidence suggests that alcohol use on social media has even stronger effects because it is more closely linked to descriptive and injunctive norms that consequently leads to a stronger impact on drinking behaviors [60]. The role of alcohol posts on social media should not be underestimated and should be incorporated in interventions that aim to decrease excessive alcohol use.

### Limitations and Future Research

Some limitations should be noted. A complication of interpreting the effects of social posts is that the audience size of posts may differ between social and nonsocial posts (more people are tagged in social posts, thereby increasing the number of people who see the post), potentially explaining part of the relationship between social posts and increased responses. However, the fact that not only the number of people but also whether someone was holding the alcoholic beverage influenced the number of likes suggests that something alcohol-specific may further increase the impact of social features on likes. To answer the questions whether and why social alcohol posts lead to more reactions with more certainty, future studies are necessary comparing social versus nonsocial alcohol posts and contrasting those with similar neutral posts and posts about other unhealthy behaviors (eg, smoking or snacking).

Another limitation lies in the fact that we coded the alcohol posts from an outsider viewpoint—coders looked at an alcohol post and decided whether it was social (if people were visible). However, another worthwhile method may be to ask participants to describe and interpret alcohol posts (in line with Hebden et al [61], for example) to see whether any differences arise between our coding and participant interpretations. For example, it is possible that although the alcohol post in Figure 2 was coded as having no person visible, when participants see this post they may infer that the poster is probably not alone while enjoying this drink. Such interpretations are an interesting avenue for future research.

### Conclusions and Potential Implications for Interventions

This study investigated the social content and social processes involved with alcohol posts on social networking sites. Findings revealed that in terms of content and processes, alcohol posts on social media are social in nature*.* Furthermore, these social aspects are related to responses to these posts: the more social elements in the post, the more (positive) reactions (eg, likes and comments). Taken together, these findings suggest that alcohol posts on social media are a part of young people’s everyday social lives, and interventions aiming to decrease alcohol posts should include a focus on the individual in a social (networking) context.

Potential implications for interventions arise from this study. First, the comparison between the participants who did and did not post about alcohol showed that, as a group, more older young adult women post alcohol content on social media. This may illustrate an important target group for future interventions addressing the posting of alcohol-related posts. Second, our findings support viewing the posting of alcohol posts as a social behavior. Therefore, attempts to reduce this behavior should not take an individualistic approach but should focus on the individual in a social context. It may be worthwhile to make people aware that they post about social gatherings in which alcohol is visible and tag others in these posts and such posts may have unintended negative consequences and should be avoided by not posting these pictures or by hiding alcoholic beverages when a photo is taken. Adolescents and young adults can be motivated to not allow tags in such posts and to stimulate others to not post alcohol posts or tag them in it. Whether and which of these tactics will be successful is an important avenue for further research.

## Abbreviations

RQ: research question
